# Assessments of health services availability in humanitarian emergencies: a review of assessments in Haiti and Sudan using a health systems approach

**DOI:** 10.1186/s13031-015-0045-6

**Published:** 2015-06-08

**Authors:** Jason W. Nickerson, Janet Hatcher-Roberts, Orvill Adams, Amir Attaran, Peter Tugwell

**Affiliations:** Bruyère Research Institute, 85 Primrose Ave, Room 308-B, Ottawa, ON K1R 6M1 Canada; Institute of Population Health, University of Ottawa, Ottawa, ON Canada; WHO Collaborating Centre for Knowledge Translation and Health Technology Assessment in Health Equity, Bruyère Research Institute, Ottawa, ON K1R 6M1 Canada; Orvill Adams and Associates, Ottawa, ON Canada; Faculties of Law and Medicine, University of Ottawa, Ottawa, ON Canada; Department of Medicine, Faculty of Medicine, University of Ottawa, Ottawa, ON K1H 8M5 Canada

## Abstract

**Background:**

Assessing the availability of health services during humanitarian emergencies is essential for understanding the capacities and weaknesses of disrupted health systems. To improve the consistency of health facilities assessments, the World Health Organization has proposed the use of the Health Resources Availability Mapping System (HeRAMS) developed in Darfur, Sudan as a standardized assessment tool for use in future acute and protracted crises. This study provides an evaluation of HeRAMS’ comprehensiveness, and investigates the methods, quality and comprehensiveness of health facilities data and tools in Haiti, where HeRAMS was not used.

**Methods and findings:**

Tools and databases containing health facilities data in Haiti were collected using a snowball sampling technique, while HeRAMS was purposefully evaluated in Sudan. All collected tools were assessed for quality and comprehensiveness using a coding scheme based on the World Health Organization’s health systems building blocks, the Global Health Cluster Suggested Set of Core Indicators and Benchmarks by Category, and the Sphere Humanitarian Charter and Minimum Standards in Humanitarian Response.

Eight assessments and databases were located in Haiti, and covered a median of 3.5 of the 6 health system building blocks, 4.5 of the 14 Sphere standards, and 2 of the 9 Health Cluster indicators. None of the assessments covered all of the indicators in any of the assessment criteria and many lacked basic data, limiting the detail of analysis possible for calculating standardized benchmarks and indicators.

In Sudan, HeRAMS collected data on 5 of the 6 health system building blocks, 13 of the 14 Sphere Standards, and collected data to allow the calculation of 7 of the 9 Health Cluster Core Indicators and Benchmarks.

**Conclusions:**

There is a need to agree upon essential health facilities data in disrupted health systems during humanitarian emergencies. Although the quality of the assessments in Haiti was generally poor, the large number of platforms and assessment tools deployed suggests that health facilities data can be collected even during acute emergencies. Further consensus is needed to establish essential criteria for data collection and to establish a core group of health systems assessment experts to be deployed during future emergencies.

## Introduction

Acute and protracted crises have grave immediate and long-term effects on population health and health systems, particularly in low-income countries. Conflicts and sudden onset disasters create disruptions in the availability of health services and exacerbate long-standing health problems, while simultaneously increasing acute morbidity and mortality. Health workers may leave, be killed, or have disappeared, health facilities may be attacked, looted, or destroyed, normal supply chains may become inaccessible, and the overall functionality of the health system is disrupted [1–3]. This disruption widens the gap between a population’s health needs and the availability of essential health services to address them [4]. Implementing effective health interventions that address the most pressing health needs in these environments requires accurate assessments of the health status of the affected population and the functionality of the health system in order to respond to gaps in health services either created or exacerbated by the crisis [5].

Despite the centrality of health data as part of a coordinated humanitarian response, significant gaps have been highlighted in the methodologies used to collect most kinds of health information in crisis settings, where a lack of appropriately trained field staff who can implement complex study designs and data collection strategies has led to serious concerns of methodological validity [6–9]. This is further complicated by logistical constraints, which may make the collection of data impossible due to security or other restrictions [10]. Beyond discussions from within the humanitarian community, the collection of scientifically valid health data during major emergencies has been the subject of considerable outside scrutiny, most notably in the conduct of mortality surveys, which have garnered significant political and public interest [11–14]. In response to these concerns from within and outside of the humanitarian response community, there has been much work done to improve the quality of needs assessments recently, including the development of the Multi-Cluster Initial Rapid Assessment (MIRA); however, evidence-based guidance is currently lacking for the development of assessment tools for systematically monitoring essential elements of the health response across the health system, including monitoring levels of health service provision in health facilities, and other health system building blocks (financing, health workforce, leadership/governance, information systems, and medical products, vaccines, and technologies) [15].

The World Health Organization identifies three areas of focus for information collection and analysis for effective humanitarian health coordination: data on the health status of the population, the availability of health resources and services, and the performance of the health system [16]. These data provide a means of quantifying the health needs of the population, gaps in the coverage and functionality of health services, and the proportionality of a response needed to address them [17, 18]. While there has been a growing interest and body of research concerning health needs assessments in emergencies, there is a more limited understanding of how needs assessment data are used in operational decision-making [19–21]. The likely reasons for a lack of evidence-based decision making are pragmatic: conducting detailed needs assessments and analyses often requires slowing down or redirecting resources from another important process. Furthermore, the task of collecting information on the activities of health partners has become more complex as the number of aid agencies responding to recent sudden-onset crises has proliferated. Following the 2010 earthquake in Haiti, for example, 44 foreign medical teams and field hospitals were deployed in the first 15 days, and within the first month there were 246 organizations registered with the Pan American Health Organization (PAHO)-run Health Cluster as providing health services in the country, which is almost certainly an underestimate of the true number of aid agencies on the ground [22, 23].

Data on the availability and functionality of essential health systems factors are essential for developing an informed perspective on needs and capacities, and for reducing duplicative or ineffective interventions. These have been noted following the 2004 Indian Ocean tsunami when reports suggested that some children may have received up to four measles vaccinations, while others received none [24] and in Haiti following the 2010 earthquake, where some areas of Port-au-Prince had duplicative services, while other parts of the city had little to no access to surgical care [25].

There is a growing body of research on the assessment of national health systems performance in low-income countries, particularly in stable development contexts. There is, however, limited practical guidance on the assessment of disrupted health systems in acute crises or as part of health systems rebuilding following conflicts and disasters [26–28]. In stable development contexts, health facilities assessments provide a useful means of monitoring the availability of and readiness to deliver health services. These assessments comprise a detailed inventory of defined health services, drugs and devices, health human resources, and other important details, often in the form of lengthy assessments [29]. The resultant product is a census of health facilities throughout the country, and the ability to identify service delivery gaps and calculate the effective coverage of essential health services.

Health facility assessments of the depth and complexity used in stable contexts are clearly inappropriate for use during crises, as the tools contain lengthy sets of indicators. The time and effort required to complete these assessments makes them impractical for situations where levels of service provision change rapidly or frequently, and where resources for conducting assessments are often scarce. To improve information management practices for monitoring the availability of health services, the World Health Organization has proposed the use of the Health Resources Availability Mapping System (HeRAMS) tool. HeRAMS was developed for use in the Darfur states of Sudan as a health facility assessment tool to monitor the availability of health services across a large, remote, and largely inaccessible area with frequent disruptions to the availability of health services. While HeRAMS appeared to have been used successfully for several years, it was unclear how comprehensively the tool measured existing standards for health action in humanitarian emergencies, how easily it could be applied in other contexts (particularly sudden onset disasters), and what other tools had been developed and used in other crises for the same purpose. To address these concerns, we conducted a field-based assessment to identify other assessment tools used to compile data on the availability and functionality of health services in disrupted health sectors, and to evaluate the comprehensiveness of HeRAMS using existing and accepted standards for humanitarian emergency response. In addition to a real-time, field-based analysis of HeRAMS in Sudan, a field mission to Haiti (where HeRAMS was not used) was conducted to locate alternative assessment tools and databases used to systematically monitor the availability of health services and other essential health systems functions in health facilities in the 2 years following the 2010 earthquake. The goal of this study was to understand how health facilities data are collected across disrupted health systems, and the strengths and weaknesses of different approaches.

## Methods

This study was approved by the University of Ottawa Health Sciences and Sciences Research Ethics Board.

Data were collected during field visits by one of the authors (JWN) to Haiti (November/December 2011) and Sudan (May 2012). These two locations were selected based on the following: most pragmatically, the study was shaped by logistical considerations of operating in difficult environments, and the resources available to support operational field research, both of which were feasible in Haiti and Sudan. Additionally, the study responded to a need to evaluate the HeRAMS tool in an operational context, rather than by way of a desk review. This review was based on an understanding of how health services were assessed during the sudden-onset disaster of the Haiti earthquake, and provided insight into lines of inquiry regarding how HeRAMS functioned as a system and process. The review of HeRAMS was essential for understanding its potential applicability in future crises, framed by an understanding of its use in Darfur, as well as by the development of other assessments for similar purposes.

Because no systematically-populated databases of correspondence or documents are known to exist following the Haiti earthquake [30] or in Sudan, a snowball sampling approach was used, enlisting guidance from 21 key informants in the field (14 Haiti; 7 Sudan) and by contacting agencies who contributed to the coordination of humanitarian assistance to identify all of the known documents of interest to this study. Key informants included current and past humanitarian coordinators from various United Nations agencies, field staff from governmental and non-governmental organizations, and local ministry of health staff. These interlocutors identified both current and previous databases and assessment tools that were in use. These informants assisted in the identification of known data or assessments that provided an overview of the functionality of health services across the existing and humanitarian health systems, both at the time of the field visits and during previous missions. At the time of both field visits, both countries were in a state of protracted emergency: in Haiti due to the cholera epidemic and earthquake recovery, and in Sudan due to the ongoing conflict, displacement, and political instability. In both settings, humanitarian responses continued to be underway and evolving, though both were largely outside of the acute phase of these crises. Any relevant documentation was obtained and reviewed; this was often located only with the assistance of key informants, rather than through known databases (e.g., Reliefweb). This approach has been reported by other authors working in complex environments [31].

All accessible databases and assessment tools that contained or collected data on the location, capacity, and functionality of health facilities and the availability of health services were collected in Haiti, and alternative or complementary databases to HeRAMS were sought in Sudan. Assessments whose purpose was limited to the collection of basic identification information that did not contribute to the monitoring of the coverage of health services, such as lists of only phone numbers, location data, or contact information, were excluded from the analysis. Individual assessments conducted by aid agencies for the purpose of planning their own response were not included, as these were both generally inaccessible and not intended to comprehensively identify all health partners or functional health facilities across large health systems.

To identify any other potential sources of information, Health Cluster Bulletins, PAHO Situation Reports, and the Consolidated Appeals Process documents produced in Haiti from January 2010-December 2011, available through ReliefWeb were reviewed [32]. In Sudan, the Consolidated Appeals Process, Humanitarian Workplans, and HeRAMS Reports for Darfur for 2010–2011 were reviewed. Any examples of health facilities assessment data, as well as any reporting of health services coverage or indicators were extracted for analysis, and informed a broader contextual understanding of the emergency response.

The included assessments were entered into data analysis software (NVivo 9, QSR International) and were evaluated for their comprehensiveness, based on two frameworks of minimum standards and indicators for humanitarian health assistance: the Global Health Cluster Suggested Set of Core Indicators and Benchmarks by Category [16]; and the Sphere Humanitarian Charter and Minimum Standards in Humanitarian Response [33].

To evaluate the comprehensiveness of the included assessments in addressing the essential features of health systems and health services delivery, a framework of health facilities assessment criteria was applied to the analysis [29]. This framework was developed for a previous study, and includes criteria representing 41 different assessment domains that correspond to the six World Health Organization health systems building blocks, (Leadership/Governance; Health Care Financing; Health Workforce; Medical Products, Technologies; Information and Research; and Service Delivery) [15]. Each of the included assessments was coded according to the domains in the framework to determine their comprehensiveness. Because few of the assessments included sufficient detail on the kinds of health services provided, the analysis was reduced to the six health systems building blocks, using the 41 domains as guiding and inclusion criteria.

A similar approach was applied for both the Global Health Cluster (GHC) Set of Core Indicators and Benchmarks by Category and the Sphere standards. The assessments were evaluated using the nine GHC indicators that correspond to health resources availability assessments (out of a total of 26 indicators), based on whether they were capable of collecting the necessary data for calculating each of the indicators. The calculation of the GHC indicators requires population estimates, which were assumed to be best measured through other methodologies, rather than through health facilities assessments [34, 35]. In our assessment, we assumed that these data existed and did not include this as an evaluation criteria, as population data would not routinely be collected during a health facility assessment.

A coding system was developed based on each of the Sphere Minimum Standards for Health Action, which correspond to 14 different areas of health priorities and standards. Because many of the Sphere standards exist as broad guides rather than specifically quantifiable measures [36], the included assessments were evaluated based on whether they contained information that could reasonably correspond to each standard, using the same approach to coding described above.

## Results

HeRAMS was the only assessment tool located in Sudan, while 8 assessment tools or databases were included from Haiti (Table 1). The results of the analysis of the health systems building blocks and Global Health Cluster core indicators are contained in Table 2, and the results of the analysis using the Sphere standards are contained in Table 3. Individual country results are presented below.

**Table 1:** Included assessments or databases of health facilities and health services availability

Haiti (*n* = 8)	Sudan (*n* = 1)
Travax Database	Health Resources Availability Mapping System (HeRAMS)
Sahana Foundation Database	
International Organization for Migration (IOM) Displacement Tracking Matrix (DTM)	
Ministère de la Santé Publique et de la Population (MSPP) Elaboration de la Carte Sanitaire, Niveau SSPE & Niveau HCR	
Pan-American Health Organization (PAHO) Haiti Health Facilities Master List	
Health Cluster 4W List	
Clinton Foundation List of Health Facilities	
Health Cluster List of Mobile Clinics

**Table 2:** Analysis of included assessments and databases using health systems indicators and global health cluster core indicators

	Haiti health facilities lists (*n* = 8)	HeRAMS (*n* = 1)
Assessment Characteristics, No. (%)		
NGO	2 (25 %)	0 (0 %)
PAHO/WHO	3 (37.5 %)	1 (100 %)
Ministry of Health	1 (12.5 %)	0 (0 %)
Other	2 (25 %)	0 (0 %)
Health System Building Blocks, No. (%)		
Leadership & Governance	8 (100 %)	1 (100 %)
Health Care Financing	1 (12.5 %)	1 (100 %)
Health Workforce	4 (50 %)	1 (100 %)
Medical Products & Technologies	4 (50 %)	0 (0 %)
Health Information Systems	4 (50 %)	1 (100 %)
Service Delivery	7 (87.5 %)	1 (100 %)
Inputs for Health Cluster Core Indicators, No. (%)		
Average population per functioning health facility	2 (25 %)	1 (100 %)
Number of HF with BEmOC/500,000 pop.	1 (12.5 %)	1 (100 %)
Number of HF with CEmOC/500,000 pop.	1 (12.5 %)	1 (100 %)
Percent of HF without stockout of a selected essential drug in 4 groups of drugs	1 (12.5 %)	0 (0 %)
Number of hospital beds per 10,000 pop.	3 (37.5 %)	1 (100 %)
Percentage of HF with clinical management of rape survivors + emergency contraception + PEP available	1 (12.5 %)	1 (100 %)
Number of health workers (medical doctor + nurse + midwife) per 10,000 pop.	4 (50 %)	1 (100 %)
Number of CHWs per 10,000 pop.	1 (12.5 %)	0 (0 %)
Number of consultations per clinician, per day	2 (25 %)	1 (100 %)

**Table 3:** Analysis of included assessments and databases using sphere indicators

Sphere indicator	Haiti health facilities lists (*n* = 8) n, (%)	HeRAMS (*n* = 1) n, (%)
Reproductive Health	3 (37.5 %)	1 (100 %)
Prioritising Health Services	4 (50 %)	1 (100 %)
Prevention of Vaccine-Preventable Diseases	1 (12.5 %)	1 (100 %)
Non-Communicable Diseases	2 (25 %)	1 (100 %)
Mental Health	1 (12.5 %)	1 (100 %)
Leadership and Coordination	8 (100 %)	1 (100 %)
Injury Care	1 (12.5 %)	1 (100 %)
Human Resources	4 (50 %)	1 (100 %)
Health Service Delivery	6 (75 %)	1 (100 %)
Health Information Management	3 (37.5 %)	1 (100 %)
Health Finances	1 (12.5 %)	1 (100 %)
Drugs and Medical Supplies	3 (37.5 %)	0 (0 %)
Communicable Disease Prevention	2 (25 %)	1 (100 %)
Communicable Disease Diagnosis and Case Management	3 (37.5 %)	1 (100 %)

### Sudan

In Sudan, the collection of health facilities data in the Darfur region was coordinated by the WHO-led Health Cluster, in collaboration with the Ministry of Health. Data were collected by local WHO staff in the Darfur states, with the support of NGOs delivering health services in the region, using HeRAMS. The Ministry of Health in Sudan was reported to have conducted additional health facilities assessments throughout the country (including in Darfur), however neither the data nor assessment instruments were retrievable for the Darfur states during the course of this study. Given this, and that no key informants were able to provide copies of this tool, the assessment was determined to be non-existent for the purposes of coordinating a humanitarian response and for this study, and thus only HeRAMS was evaluated.

The HeRAMS platform collected data corresponding to five (83.3 %) of the six health systems building blocks, neglecting only the collection of data on the availability of medical products and technologies. HeRAMS was also capable of providing data for calculating seven (77.7 %) of the GHC indicators, and these indicators were reported in various HeRAMS reports that were reviewed and which are publicly available. HeRAMS collected data corresponding to thirteen (93 %) of the Sphere standards.

HeRAMS frequently uses broad categories of health services, rather than specific functions of these health services, making nuanced assessment potentially problematic. For example, indicators such as “basic laboratory” or “antenatal care” are comprised of many separate services that are difficult to assess comparatively when one or more sub-services or functions is absent.

### Haiti

8 assessment instruments were located in Haiti that met the inclusion criteria. 1 additional assessment tool was excluded (the MSPP NGO Registration Tool – Version 2011) and 1 database (the Health Cluster list of Cholera Treatment Centres and Cholera Treatment Units), as they did not meet the inclusion criteria as both contained only limited information on the nature of the international organizations’ work or the location of cholera treatment centres, respectively. This resulted in 8 assessment instruments being included in the analysis. Other, more narrow, needs assessments of individual health facilities were conducted by many NGOs, particularly during the early stages of the emergency, but these were not intended to systematically populate a list of health facilities or health services and were not included.

A review of Health Cluster Bulletins and the Consolidated Appeals Process documents in Haiti did not locate any additional databases or assessments of health services. Furthermore, the review of these documents did not locate any data or results of any assessments of the effective coverage of health services in the country. One Health Cluster Bulletin from March 15, 2010 noted a study was to be conducted by the International Rescue Committee and Management Sciences for Health to identify gaps in primary health care coverage. Attempts to locate this study by contacting both agencies were unsuccessful, and no key informants made reference to it.

Following the 2010 earthquake, health system-wide data on the location, functionality, and services provided by health facilities were largely collected by NGOs or PAHO/WHO. Within these databases, the scope and quality of the data collected varied significantly, with some assessments collecting only data on the physical location and functionality (operational or non-operational) of the health facility, while others collected detailed data on the numbers of beds and health workers present, as well as the health services provided.

The median number of health systems building blocks assessed in each of the tools was 3.5 (range: 1–5). All 8 contained data corresponding to Leadership and Governance by way of identifying the lead agency providing services in the hospital and generally the ownership of the facility. The second most frequently included indicators corresponded to Service Delivery, and were included in 7 (87.5 %) of the assessment tools and databases. Data corresponding to the rest of the building blocks were less consistently included. The content of these assessments varied significantly, ranging from the collection of only the presence or absence of health services delivery without further explanation, to fairly comprehensive assessments of the presence of medical subspecialties. Many of the included assessments utilized aggregate assessments of health services, which limited the ability to clearly identify their individual components. For example, assessments that included broad groupings of health services into one indicator such as “obstetric care” did not allow for detailed analysis of the comprehensiveness of these services, such as whether these services met the criteria for Basic Emergency Obstetric Care (BEmOC) or Comprehensive Emergency Obstetric Care (CEmOC), or neither [37, 38].

Health Workforce data (such as the number of health professionals present in a health facility) were collected in 5 of the assessments (62.5 %), though some contained aggregate indicators such as the number of nursing or surgical teams present, rather than the actual number of individual health workers. Few of the included assessments collected data that corresponded with the remainder of the health systems building blocks. Health Information Systems (such as an early warning system for communicable diseases) and Medical Products & Technologies (such as drugs or diagnostic imaging devices) data were included in only 4 (50 %) of the included assessments, and Health Care Financing (such as whether user fees were charged, or who was financing the facility) was only included in one (12.5 %) assessment.

The median number of the nine Global Health Cluster Core Indicators that could be calculated by each of the included Haiti assessment tools and databases was 2 (range: 0–5). None of the databases or tools was capable of providing the necessary inputs for calculating all of the core indicators. Of the individual indicators, the most frequently calculable was the number of health workers (included in 4, or 50 %, of the assessments), followed by the number of hospital beds (included in 3, or 37.5 % of the assessments). The average population per functioning health facility and the number of consultations per clinician, per day could both be calculated by 2 of the assessments, while the remainder of the indicators could only be calculated by 1.

The eight Haiti assessments covered a median number of 4.5 (range 2–12) of the 14 Sphere standards for health service delivery.

## Discussion

Major emergencies present a paradoxical opportunity: on the one hand, disasters and conflicts result in the loss of lives and critical infrastructure, and disrupt the lives of large numbers of people, often in countries where basic social services are already fragile. On the other, emergencies have the potential to rapidly mobilize large amounts of technical, material, human, and financial assistance that may not otherwise have been committed. In order to optimally capitalize on this mobilization of resources, humanitarian agencies must rapidly assess what is needed, what is already in the process of being provided, and what is duplicative or ineffective, using methods that are appropriate in the context and through an approach that is trusted by all partners involved. This “window of opportunity” has been described by others, and although conceptually appealing, appears unlikely to immediately and rapidly result in major health system reforms within a short period of time even as the emergency gives way to rebuilding [39]. What does seem clear, however, is that an opportunity to rapidly scale up the availability of essential health services exists, and doing so effectively requires a sound understanding of the healthcare environment and the availability or absence of these services.

We believe that this study consists of the first field-based evaluation of health services and health facilities assessment instruments conducted during humanitarian emergencies. In Haiti while there was a plurality of assessments and databases created following the earthquake, there was not a centralization of data collection tools or information management, which likely impeded the effective analysis of the adequacy of the response or the identification of needs. In Darfur, where only one system (HeRAMS) exists for the central management of the availability of health information, analyses were performed and decisions were able to be made based on these analyses. Clearly, the outright comparison of these emergencies is not possible: Haiti was a relatively recent, sudden-onset disaster at the time of data collection, whereas the Darfur crisis was a protracted complex humanitarian emergency that had established operations and centralized information management practices, developed over nearly a decade. Given that WHO has expressed a desire to expand the use of HeRAMS in future emergencies (and already has since the completion of this study), the comparison of the comprehensiveness of different systems in different emergencies is warranted. When examined collectively and comparatively, the results from the two countries present several important findings with regard to health systems data collection in humanitarian emergencies.

The results from Haiti demonstrated that even during the initial stages of the emergency response, there were several agencies collecting data on the location, functionality, and capacity of existing health facilities and incoming foreign medical teams across the health system. In addition to this, many responding NGOs were conducting their own initial rapid assessments, and the Multicluster Initial Rapid Assessment (MIRA) was also deployed to establish both a general assessment of humanitarian needs and specific needs within various sectors. While many of the initial rapid assessments (and agency-specific assessments) were conducted by expert humanitarian assessment specialists, many of the assessments and databases included in this study were collected by specialist agencies and volunteers, many of whom could reasonably be presumed to have been part of the crisis mapping community that emerged following the earthquake [40]. Groups such as OpenStreetMaps and Ushahidi provided vital assistance in the mapping of infrastructure, including health facilities, while other organizations such as the Sahana Foundation and Travax were actively collecting and disseminating their own data on health facilities. Several platforms such as Google’s Resource Finder and HealthMap also utilized many of these databases to create maps of the location of health facilities. It is not known to what extent these platforms were used by responding agencies, however the data were routinely updated and the platforms were easily accessible, suggesting that the potential for their use was significant. The important caveat to this statement is, however, that these systems functioned only with the availability of mobile phone and internet access, which was initially unavailable following the earthquake. Similarly, in Darfur, HeRAMS, whose data collection tool and platform used a Microsoft Excel spreadsheet, also collected information from remote health facilities that lacked reliable internet, electricity, and mobile phone access, and relied on paper-based versions of the assessment for these regions. This highlights the need for an adaptable approach to data collection, inclusive of both paper-based and electronic means of collecting and sharing data.

The collection, analysis, and mapping of large amounts of health facilities data following the Haiti earthquake suggests that the rapid deployment of information gathering platforms is technically feasible in crisis settings. Furthermore, it suggests that large volumes of data can be collected, even under difficult settings, particularly using online platforms when these services are available. This is further exemplified by HeRAMS in Sudan, where data on health facilities in remote locations as well as in semi-urban camp settings are collected under precarious security constraints. While several platforms existed and were simultaneously operational in Haiti, they lacked a central coordinating mechanism for ensuring their dissemination, uptake, and validation.

What is significant from the Haiti experience is that there was not an absence of data, but rather an abundance of it, collected in different formats, on different platforms, using different indicators, and with varying degrees of comprehensiveness. Some platforms used primary, on-site data collection, others used secondary data sources, while others appear to have used a blended approach. The focus, here, appears to have been to rapidly assemble a list of both previous health facilities and new (i.e., field hospitals) health services, with varying degrees of success. Many of the tools used indicators that appeared to correspond to specific priorities, but were ultimately likely not of broad value, for example the number of ventilators present in individual health facilities.

This work highlights the need for coordinated health systems information management as part of humanitarian coordination structures, which is also a finding of the PAHO-commissioned review of the Haiti earthquake health response [30]. Despite eight different attempts at assessing and mapping health facilities data following the Haiti earthquake, the results of this study suggest that none provided a comprehensive assessment of emergency health services in a manner that corresponded with existing standards for humanitarian response. While debate has been raised concerning the measurability and appropriateness of the Sphere Standards, they provide at least one mechanism for monitoring essential services across disrupted health systems. Regardless of the appropriateness of their quantitative benchmarks, it seems self-evident that if the maintenance of a minimally functional health system is a priority, attention should be paid to monitoring the availability of essential services such as mental health, pharmaceutical supply chains, and emergency obstetric care, among others. That these data were not part of the included assessments does not mean that they did not exist or that other, more detailed assessments were not conducted; what this suggests is that these data were not easily consolidated into a system capable of providing information on the availability of a range of health services and functions, which is something that WHO has sought to improve through the more widespread use of HeRAMS and other tools.

The data that were collected in Haiti do not appear to have been used to generate analyses of the adequacy of the response at any time in the nearly two years examined by this study, through the calculation of the nine Global Health Cluster core indicators for health resource availability, nor could any of them comprehensively have done so with the data they collected. Evidence from Health Cluster bulletins suggests that information on the availability and functionality of health facilities was being collected and disseminated by participating agencies. Several sources (key informants, document reviews) have noted that this information was being shared and that many responding agencies were likely aware of the medical capacity within the response. However, the systematic documentation of this appears to have been lacking.

This study included one dataset modeled on the 3W tool, which is often used to quickly assemble a list of basic data (who, what, and where – 3W), and which in Haiti was deployed by the United Nations Office for the Coordination of Humanitarian Affairs (UNOCHA). This assessment contained information on health activities, but fell short of the comprehensiveness of the other more spontaneous datasets examined, in large part due to the nature of the 3W tool, which is not generally intended to be used as a health facility assessment tool or census. It was included, however, because in Haiti, the 3W contained data that attempt to determine the availability and functionality of health services, asking questions about the availability of health facilities and the prevalence of health conditions in a manner consistent with the inclusion criteria.

Had the technical prowess and reach of the volunteer mapping communities been matched with the health systems expertise of the more established humanitarian health and United Nations agencies, the resultant product may have been a more coordinated, flexible, centralized information management system. However, this synergy did not occur and the good and the bad were neither scaled-up, nor culled. There were calls for such standardization of data collection (one online wiki created to coordinate volunteer mapping efforts calls for the creation of a central call center for reporting health facilities data, using undefined “PAHO standards” [41]), though this consolidation of information appears to have not occurred. Arguably, the response suffered from more than a lack of public health and health system information in the coordination of the emergency [19]; however, others have also noted this information as being an absent, yet necessary, component in the response [30].

Comparatively, in Sudan, where health services availability data collection is embedded within the WHO-led Health Cluster, a reasonably organized system for collecting, analyzing, and disseminating this information exists. The success of this system appears to lie in the enabling role of the Health Cluster in ensuring that physical and human resources exist to centralize and coordinate data collection and dissemination: WHO field staff and Ministry of Health officials in each state ensure data are collected from NGO and government-run health facilities and is sent to data management coordinators in Khartoum who process, analyze, and disseminate findings on a quarterly basis. While there is reason to be cautiously optimistic of this system (data are collected under precarious security circumstances within a disrupted health system, leading to legitimate concerns of data quality and accuracy) it provides a reasonable model for the centralization of health facilities data collection and analysis in disrupted health systems in emergency settings. Further discussion of these challenges is provided in a subsequent manuscript.

These results should not be taken to suggest that a one-size-fits-all approach is what is needed. Quite clearly, during acute crises there is a need for rapid assessments comprised of qualitative and quantitative, primary and secondary data in order to make decisions that are reasonably well-informed [26]. Given the complexity of the response in Haiti (and elsewhere), and the rapid evolution and turnover of health services, it would be near impossible to implement an overly comprehensive or complex health information system during the acute phase of an emergency. However, the results of this study demonstrate that the rapid collection of information on the availability of important health services is possible using broad indicators of services (e.g., “surgical care” or “mental health services”). What is needed is a common operating framework to guide assessments and to tailor their complexity across different phases of the emergency, ensuring continuity and comprehensiveness in the domains assessed, but responding to the realities of what data are feasible and useful to collect during emergencies. More complex assessments and tools (such as the WHO’s Service Availability and Readiness Assessment) likely have a place outside of the emergency, in the recovery stage, when the situation stabilizes and some equilibrium is reached within the health system. The determination of this threshold requires careful scrutiny, and the use of aggregate indicators during the acute emergency response should be guided by a need to gradually evolve toward more detailed assessments and datasets as the emergency stabilizes. Attempts to standardize the definitions of essential health services should be viewed as part of the professionalization of foreign medical teams, where the composition and essential services that these teams provide are currently being determined [42, 43].

The lack of definition of essential health services requires urgent attention in order to establish minimum standards for their delivery and the development of indicators to monitor them. Standardized definitions exist for some groups of health services (such as emergency obstetric care [37] and the Minimum Initial Services Package for reproductive health [44]) and monitoring criteria have been proposed for others, such as surgical care [45], though others must be developed to address the range of essential health services. The analysis of various assessments for this project was directly hampered by an inability to properly discern the specificity, adequacy, and quality of broad groups of vaguely-defined health services – it would logically follow that decision-making in the field would similarly face such uncertainty when given the same data [3].

The reporting of these results suffered from similar shortfalls and challenges in consolidating useful information, particularly in the Haiti response. While several examples of reporting of numbers of functioning health facilities in Health Cluster Bulletins and Situation Reports from Haiti were located, none provided an analysis of the adequacy or coverage of health services. Similar findings have been identified concerning the reporting of epidemiologic data in Haiti [46], and other reviewers have noted that health systems indicators were seldom analyzed before the earthquake, and “least of all under the chaotic conditions and extreme pressure for immediate action” [30].

Recognizing that under emergency situations, information exists in a tenuous balance between having too little to make informed decisions, and being overwhelmed by superfluous data, it seems evident that consensus is needed on which kinds of information are most useful for coordinating the health response to an emergency and how best to go about collecting and analyzing it. From a health services and resources management perspective, what is needed is a specific and timely assessment of which resources are already available and functional, which ones are in the process of being made available, and what services and resources are lacking and could be addressed through the mobilization of national or international resources.

While there may be a place for specific assessment tools, such as HeRAMS, in situations where no health information system exists or where these systems are non-functional, this may not apply in all environments, particularly when reasonable information systems already exist. A potentially more useful focus could include updating and extracting core data and analyses (such as the Global Health Cluster core indicators) from existing information systems, rather than aiming to duplicate or replace them, unless they are not fit for the purpose of rapid analyses during emergencies. In either case, the availability of baseline data are essential components for understanding the initial situation and for projecting potential health risks as a result of emergencies.

Conducting appropriate analyses requires the development and mobilization of professionals and NGOs with expertise in the area of health systems in emergencies, similar to what has been advocated for in the conduct of field mortality surveys [9]. Such an approach would allow for the collection of relevant data, the conduct of appropriate analyses to obtain useful outputs, and the collaborative development of a strategy for strengthening the country’s health information system (rather than duplicating or replacing it) as part of the recovery.

### Limitations

This study has several important limitations that are common to the conduct of field-based research in crisis settings [47]. Owing to the nature of humanitarian responses, characterized by fragmented information management and high staff turnovers, limited institutional memory exists, thus creating the possibility that some data may have been missed. This limitation was likely reasonably mitigated by interviewing 21 key informants in the field, as well as by contacting former humanitarian staff that were able to provide additional insight and access to documents. The results of key informant interviews will be published in a separate manuscript. This study does not, however, include those assessments that were conducted by individual responding agencies and which were shared as part of the initial information management strategy during the early days of both responses. While these assessments would have provided some insight into the functioning of the health systems in which they were deployed, these assessments were not intended to form a database of health facilities or to be used to generate health systems-level analyses of interest to this study (such as a trends analysis of the availability of certain services). Thus, we feel justified in not including them.

This study relies on evidence collected in two different countries and types of humanitarian emergencies: Haiti, whose fragmented health system has been disrupted by an earthquake, an influx of foreign assistance, and a cholera epidemic; and a complex humanitarian emergency in the Darfur region of Sudan, where populations are displaced and health care providers are routinely challenged by insecurity, a lack of resources, and a precarious public health environment. While there is a need to conduct and share research on humanitarian interventions, comparisons from one context to another require careful consideration [48]. The two emergencies studied here are not controlled, comparable environments; however, few settings for humanitarian intervention are, and this is a considerable challenge for public health researchers in this field [49]. Results (including those of this study) are not always easily transferrable from one context to another [50], further supporting calls for the development of specialist health systems experts in emergencies who can guide the implementation of complex study designs and analyses in austere settings. Fundamentally, the goal of this study was not to compare one context to another; it was to document the tools and methods used and to provide an analysis of their quality and comprehensiveness.

Detailed cost information was unavailable to us throughout the course of this project, making it impossible to provide reliable cost comparisons for the implementation of these information systems. Furthermore, we were unable to determine their impact on the financial and programmatic sustainability of health systems interventions, though the impact on resource allocation and intervention planning is discussed in a subsequent manuscript.

Finally, the timing of the assessments presents a difficult variable to control for. In Haiti, assessment tools and databases were included from the time of the 2010 earthquake to the time of the field visits; in Sudan, only one tool was included (and in use), and the field visit took place nearly a decade into a protracted emergency. Further, we were unable to make reliable estimates of the timeframe for which the data were collected in Haiti. Thus, it is likely that comparisons were made between tools developed during the initial days or weeks of the earthquake response and those developed much later. Given that even when the assessments are viewed individually their inclusion of the standards of interest was quite low, we do not believe this to have material significance affecting our conclusions.

## Conclusion

The results of this study suggest that the assessment of the availability and functionality of health services and facilities is technically feasible in emergencies, even under difficult circumstances. Furthermore, this research identifies several examples where groups and organizations collected these data during a sudden onset disaster, and established parallel health information systems *de novo*. While these assessments did exist in Haiti, serious concerns were uncovered related to the quality, analysis, and sharing of their data, with little indication that essential health systems indicators were calculated.

Further work is needed to develop consensus on assessment criteria for health resources and services availability assessments in crisis settings. This should closely mimic packages of essential health services and the ongoing efforts to standardize and professionalize foreign medical teams. Streamlined data collection tools using evidence-based and agreed-upon essential datasets should be developed and deployed, reflective of broad health system considerations, minimum standards in humanitarian response, and essential health system analytics for coordinating a humanitarian response and health system recovery. Beyond the selection of indicators at the facilities level, guidance is needed for utilizing and analyzing health systems benchmark data collected prior to the emergency, and for calculating the effective coverage of essential health services and core health systems indicators.

There is a need for further research that uses a prospective approach to systematically collect individual agency health facility assessments, as this would provide additional insight into the comprehensiveness of data being collected as part of the initial decision-making in emergencies. This was outside of the scope of this project, but would provide further insight into the prioritization of health services.
